# Cross-species transmission potential between wild pigs, livestock, poultry, wildlife, and humans: implications for disease risk management in North America

**DOI:** 10.1038/s41598-017-07336-z

**Published:** 2017-08-10

**Authors:** Ryan S. Miller, Steven J. Sweeney, Chris Slootmaker, Daniel A. Grear, Paul A. Di Salvo, Deborah Kiser, Stephanie A. Shwiff

**Affiliations:** 1 Center for Epidemiology and Animal Health, Veterinary Services, Animal and Plant Health Inspection Service, United States Department of Agriculture, Fort Collins, Colorado United States; 2 National Wildlife Research Center, Wildlife Services, Animal and Plant Health Inspection Service, United States Department of Agriculture, Fort Collins, Colorado United States; 3 National Wildlife Health Center, United States Geological Survey, Madison, Wisconsin United States

## Abstract

Cross-species disease transmission between wildlife, domestic animals and humans is an increasing threat to public and veterinary health. Wild pigs are increasingly a potential veterinary and public health threat. Here we investigate 84 pathogens and the host species most at risk for transmission with wild pigs using a network approach. We assess the risk to agricultural and human health by evaluating the status of these pathogens and the co-occurrence of wild pigs, agriculture and humans. We identified 34 (87%) OIE listed swine pathogens that cause clinical disease in livestock, poultry, wildlife, and humans. On average 73% of bacterial, 39% of viral, and 63% of parasitic pathogens caused clinical disease in other species. Non-porcine livestock in the family *Bovidae* shared the most pathogens with swine (82%). Only 49% of currently listed OIE domestic swine diseases had published wild pig surveillance studies. The co-occurrence of wild pigs and farms increased annually at a rate of 1.2% with as much as 57% of all farms and 77% of all agricultural animals co-occurring with wild pigs. The increasing co-occurrence of wild pigs with livestock and humans along with the large number of pathogens shared is a growing risk for cross-species transmission.

## Introduction

Diseases transmitted between humans, wildlife, and domestic animals are increasingly challenging public and veterinary health systems^1, 2^. In North America, it is estimated that at least 79% of reportable domestic animal diseases have a putative wildlife component associated with the transmission, maintenance, or life cycle of the pathogen and at least 40% are zoonotic^3^. Similarly three-fourths of all emerging infectious diseases (EIDs) of humans are zoonotic with most originating from wildlife reservoirs^4, 5^. Therefore, diseases that arise from the livestock–wildlife interface are of paramount importance and must be an area of focus for public and veterinary health systems^6^. Despite this importance cross-species transmission is one of the least studied aspects of disease ecology^7, 8^.

Wild pigs (*Sus scrofa*), that include feral domestic pigs (*Sus scrofa domestica*), Eurasian wild boar (*Sus scrofa linnaeus*), and hybrids between the two, are the most abundant free-ranging, exotic ungulates in North America^9^. Recently, wild pigs in North America have become of increasing concern as a potential veterinary and public health threat for cross-species transmission^9, 10^. Research and policy addressing wild pig disease has received increased attention in recent years^9–11^. This is driven, in part, by substantial range expansion, increasing ecological and agricultural damage, and increased involvement of wild pigs in disease transmission^9^. In North America, wild pigs have expanded their range to at least 41 states in the United States and three provinces in Canada since the 1960s^9, 10, 12^ and recent modeling indicates that their potential range may be far greater^13^.

In some parts of the world, wild pigs have been identified as an important reservoir for epidemic diseases, such as classical swine fever virus and African swine fever virus, that have the potential for serious socio-economic consequences^14–16^. These diseases, often termed transboundary animal diseases, can cause high morbidity and mortality in susceptible animal populations constituting a threat to national economies^17^. The cost of an outbreak of foot and mouth disease (FMD) involving wild pigs is estimated to range from USD$11.9 million to USD$5.8 billion^18, 19^. In addition disease risks posed by wild pigs to other domestic animals (i.e. cattle) are increasingly identified^9, 10, 20–22^. The potential for disease outbreaks to impact international trade may also be important^23^.

In addition to agricultural impacts, wild pigs are associated with a diversity of public health issues. Wild pigs have been implicated in the transmission of zoonotic viruses such as hepatitis E virus (HEV)^24^, trichinellosis^25, 26^, swine influenza virus^27^, and Japanese encephalitis virus^28^. In addition to direct transmission, wild pigs have been identified as a contributor to O157:H7 *Escherichia coli* contamination in watersheds^29^. Interest in the role that wild pigs may play in foodborne illness has also increased after recent outbreaks of *Salmonella* spp. in spinach and other leafy greens were traced back to farms in areas with wild pig populations^29, 30^.

The threats posed by diseases in wild pigs have been recognized in North America as well as globally for some pathogen-host systems^9^. A recent evaluation of 80,000 publications addressing wildlife-livestock diseases found that only 18% of the publications addressed the domestic swine interface and that this may be an important knowledge gap given global increases in swine production^31^. While there have been numerous system specific studies investigating the role of wild pigs in pathogen transmission these studies are primarily limited to diseases of concern for domestic swine production^14, 16^ or human health^25, 26^. As a result there is not currently an assessment across all economically important pathogens known to infect swine (domestic and wild) and the potential transmission of these pathogens between wild pigs, livestock, poultry, wildlife, and humans. Here, our objectives are three fold. First, we identify economically important pathogens (bacterial, viral, and parasitic) that are potentially shared between wild pigs, livestock, poultry, cervids, and humans. Second, we evaluate the reported prevalence of these pathogens in North American wild pig populations to assess any potential gaps in knowledge. Third, to illustrate the importance of disease risk management, we investigate the number of farms potentially at risk in the United States.

To achieve these objectives we used a common risk identification methodology to identify wild pig pathogens that can be shared between livestock, wildlife, and humans by evaluating susceptibility to these pathogens^1, 3, 32, 33^. We then used these data describing pathogen susceptibility by species to develop transmission potential networks that describe the potential for pathogen sharing between species^8, 34^. Network metrics were used to identify species that had the highest potential for sharing of pathogens and identify pathogens that were most common across species. We identify gaps in knowledge required to inform surveillance, risk assessments, scientific studies, and risk mitigations for diseases of wild pigs and provide a discussion of these in the context of wild pig range overlap with agriculture in the United States.

## Methods

### Assessment and identification of shared pathogens

First we considered 84 World Organisation for Animal Health (OIE) terrestrial pathogens that were listed beginning in 2013 (bee diseases were excluded)^35^. Each of these 84 pathogens was evaluated using the published literature to determine its reported ability to infect swine (wild and domestic), cattle, sheep, goats, horses, poultry, cervids (North American deer and elk species only), and humans. A priori we identified and used nine susceptibility categories to characterize the outcome of infection in each of these host species (Table 1). Using these susceptibility categories the scientific literature was reviewed for each pathogen and based on this literature each host was assigned to the a-priori categories. A detailed description of the search criteria used are included in the supplemental. The final set of classified pathogens by host was then reviewed independently by five veterinary epidemiologists to achieve consensus based on the supporting evidence for each assigned category (details also provided in the supplemental material). This independent review reduced potential bias that maybe associated with the literature search. Where possible, we used literature to confirm whether wild and domestic swine were equally susceptible to pathogens. When literature was unavailable to discern any differences between wild and domestic swine (the case with most pathogens) we assumed that domestic swine and wild pigs were similarly affected. We summarized these data to describe the number of pathogens each species was susceptible.

**Table 1:** Susceptibility categories used to describe infection in the host species.

Category	Code	Description
Clinical	C	Capable of developing clinical disease but can also be subclinical in some circumstances.
Subclinical	SC	Can be infected but does not develop clinical disease.
Affected	A	Species group is known to be susceptible (including seropositive) however it is unclear if they become clinical or subclinical hosts.
Occasional	O	Occasionally reported, but is rare or atypical in species group.
Uncertain	U	Some evidence suggests the species may be affected; however scientific evidence is currently unclear, lacking, or anecdotal.
Experimental	EX	Species group can become experimentally infected however natural infection is unknown or not reported.
Definitive Host	DH	Species group is considered the definitive host for the parasite.
Intermediate Host	IH	Species group is considered the intermediate host for the parasite.
Dead-end Host	DEH	Species group is considered a dead-end host for the parasite.

Categories were established a-priori and used to denote the potential impact in each of these species based on available scientific literature.

### Transmission potential networks

To investigate the species (hosts) and pathogens with the greatest potential to be involved in transmission we determined the degree of association among hosts with “transmission-potential networks” (TPN), where hosts were network nodes (swine, sheep, goat, cattle, cervid, poultry, equine, human) that were connected via edges defined by similarity in pathogen susceptibility^7, 34^. Thus edges are not equivalent to networks based on contact patterns. Edges in our transmission networks depict the potential for transmission between host species based on known etiology and host range for the pathogen rather than pathogen co-occurrence in space and time^34, 36, 37^. We define transmission potential to mean the likelihood that a given host species group will infect another species group, relative to other species in the network, based on species susceptibility to the pathogen. Thus, connected species form part of the same transmission chain^7, 34, 37^. Using methods similar to Pilosof, *et al*.^34^ we generated TPNs for two general cases, 1) host pairs were both clinically susceptible and 2) host pairs were clinically or subclinically susceptible. For the first case, TPNs defined edges if two host types were clinically susceptible to the same pathogen and were constructed for all pathogens, and separately for bacterial pathogens, viral pathogens, and parasitic pathogens resulting in four TPNs. For the second case, TPNs defined edges if two host types were clinically or subclinically affected by the same pathogen and were also generated for all pathogens, bacterial pathogens, viral pathogens, and parasitic pathogens resulting in four TPN.

The structural characteristics of these networks were evaluated using both edge and node level statistics. Edge weights in the TPNs where calculated for bacterial, viral and parasitic pathogens using the Jaccard index^38^, assuming a positive relationship of pathogen infections shared by species and the likelihood that a pathogen would infect them both. Thus, an edge received its minimum value of zero when the species did not share any pathogens and its maximum value of 1 when the pair of species was susceptible to the exact same pathogens. Index values closer to 1 indicate greater potential for transmission of pathogen types while values close to zero indicate no or limited potential transmission.

Eigenvalue centrality (EC) was used to quantify the importance of a host species (node) in terms of promoting pathogen transmission potential among all host species. With EC, a species group’s importance is increased when it has more connections to other species that are themselves important^39^. EC thus enables quantification of the transmission potential of a species group among all species in the network^40, 41^. We also generated node level statistics for individual pathogens to evaluate the relative importance of individual pathogens in the networks. We used normalized degree centrality (DC) and EC metrics among the TPNs defined by each group of pathogens (bacterial, viral, parasitic and all pathogens considered together). DC increases as more species are susceptible and received its maximum value of 1 when all species were susceptible (i.e. clinical or subclinical depending on the network) to the pathogen^42^. EC for pathogens can be interpreted in the same way as host species.

### Assessment of current status of pathogens in North America

To generate data describing the current status of OIE listed pathogens in wild pigs in North America, we developed a method to sample from the scientific literature. Our approach is based on PRISMA (Preferred Reporting Items for Systematic Reviews and Meta-Analyses) method of systematic literature review^43, 44^. Our objective was not to identify all papers reporting pathogen findings but rather to generate a representative sample that could be used to determine pathogens that have existing surveillance studies. To achieve this objective, first we used keywords to search three databases (PubMed, Scopus, and Web of Science) for papers reporting surveillance results, pathology, and case reports for wild pigs for any pathogen^43, 45, 46^. We confined our search to the literature published in English since 1900. All scientific peer reviewed literature describing any wild pig pathogens in North America was considered eligible (details regarding the search criteria are included in the supplemental material). We assumed that these papers represented the known status of pathogens in swine in North America. Once all relevant sources were identified and retrieved, we reviewed each paper to ensure relevance. The numbers of papers reporting pathogen findings in wild pigs were tallied by pathogen to determine variability in known pathogen occurrence in wild pigs. We report the number of studies and the range of reported prevalence for pathogens in the North America.

### Assessment of farms and rural populations potentially at risk

To illustrate the potential risk to agriculture and humans resulting from exposure to wild pigs, we examined the co-occurrence of wild pigs, farms, and rural human population. Because data describing the distribution of wild pigs is not available for Canada and Mexico our analysis was restricted to the United States. A measure of the annual co-occurrence was developed using three data sources. Data reporting the number of farms by agricultural commodity and county was compiled from the National Agricultural Statistics Service (NASS) Quick Stats database^47^. We restricted our investigation to the livestock commodities (species) listed in Table 2 (i.e. domestic swine, cattle, sheep, goat, cervids, equine, and poultry). The county-level number of farms is available at a national scale for 2002, 2007, and 2012. For completeness, we included rural human population as a proxy for potential human-wild pig interaction. County-level estimates of rural human population are available from the 2010 census^48^. The county level distribution (presence/absence) of wild pigs, were compiled from the Southeast Cooperative Wildlife Disease Study (SCWDS)^49^. The SCWDS data represent the known distribution of wild pig populations from 1982 until present. These data were merged to generate a database describing at the county-level the number of farms, rural human population size, and the presence or absence of wild pigs. We used only the 2010 census because it was closest to the mid-point of the wild pig data and changes in rural populations were small (mean = 0.29%) and bounded zero (range −0.07 to 0.74%) from 2012 to 2013 providing a good approximation of rural populations^50^. The national proportion of farms and rural populations co-occurring with wild pigs was then calculated for the years 2002, 2007, and 2012. We estimated the increase in the number of farms and human populations co-occurring with wild pigs using linear regression.

**Table 2:** Susceptibility of seven host species to OIE listed swine pathogens.

Pathogen	Status	Wild pig Prevalence	Prevalence Study	Wild Pig	Domestic Swine	Cattle	Sheep	Goat	Poultry	Cervid	Equine	Human	Supporting Citations
**Bacterial**													
*Bacillus anthracis*	Yes			C	C	C	C	C	C	C	C	C	103–108
*Brucella abortus*	Yes	35%	109	C	C	C	C	C		C	C	C	104, 105, 110
*Brucella melitensis*	1999				C	C	C	C			C	C	105, 107, 108
*Brucella suis*	Yes	0–68.8%	20, 109, 111–114	C	C	SC	O	O			C	C	107, 108
*Coxiella burnetii*	Yes	50%	115	A	A	C	C	C	A	A	A	C	106
*Ehrlichia ruminantium*	NR					C	C	C		EX			108, 116
*Francisella tularensis*	Yes	1.3%	117	C	C	C	C	C	U	A	C	C	104, 107, 108, 118
*Leptospira* ^†^	Yes	8–87%	119, 120	C	C	C	C	C		C	C	C	104, 105, 121
*Mycobacterium avium*	Yes			C	C	C	C	C	U	C	EX	U	104–106
*Mycobacterium bovis*	Yes	2–85%	122, 123	C	C	C	C	C	U	C	C	C	1, 3, 104, 105, 124
*Pasteurella multocida*	Yes				C	C	C	C		C	C		107, 108, 125
**Viral**													
African swine fever virus	NR			C	C								107, 108
*Alcelaphine gammaherpesvirus* ^†^	Yes				C	C	SC	SC		C			104, 105, 108
Bluetongue virus	Yes					SC	C	C		C		O	108
*bovine herpesvirus 1*	Yes				C	C		C		A			104
Bovine viral diarrhea virus	Yes	0%	119		C	C	SC	SC		SC			104, 105, 126, 127
Classical swine fever virus	2015	0%	128	C	C								104, 107, 108
Crimean-Congo hemorrhagic fever virus	NR				SC	SC	SC	SC			SC	C	108
Eastern equine encephalomyelitis virus	Yes	16.5%	129, 130	C	C	C	C	C	C	C	C	C	131–133
*Ehrlichia ruminantium*	NR					C	C	C		EX			108, 116
Epizootic hemorrhagic disease virus	Yes					C	EX			C			108, 134, 135
Foot and mouth disease virus	1947			C	C	C	C	C		C		C	107, 108
Influenza (avian) virus	Yes	1–14.4%	27, 73	C	C	A	SC	SC	C	SC	C	C	136–141
Influenza (equine) virus	Yes				C	EX			A	U	C	C	142, 143
Japanese encephalitis virus	NR				C	SC	SC	SC	SC		C	C	105, 107, 108, 131, 132, 144, 145
Nipah virus	NR				C		U	C			C	C	105, 107, 108
Peste des petits ruminants virus	NR				EX	SC	C	C		EX			107, 108, 146
Porcine epidemic diarrhea virus^†^	Yes				C								137, 147
Porcine repro. and resp. synd. virus^†^	Yes	1–3%	76, 148, 149	C	C								76, 104
*Rabies lyssavirus*	Yes				C	C	C	C		C	C	C	107, 108
Rift Valley fever virus	NR				A	C	C	C	A			C	108, 150
*Rinderpest morbillivirus*	NR				C	C	C	C					151, 152
*Suid herpesvirus 1*	Yes	7–61%	14, 77, 113, 153	C	C	C	C	C	U	C	O		104, 105, 107, 108
Swine vesicular disease virus	NR				C								107, 108
Transmissible Gastroenteritis Coronavirus	Yes	0%	148, 154	C	C								104
Venezuelan equine encephalomyelitis virus	1971				C	C	C	C	SC		C	C	107, 108
Vesicular stomatitis virus	Yes	0–100%	57, 155, 156	C	C	C	C	C	A, EX	A	C	C	104, 107, 108, 157
West Nile virus	Yes	16.1–32.1%	158	SC	SC	SC	C	SC	C	C	C	C	108, 159–162
**Parasitic**													
*Echinococcus sp*.	Yes			C, IH	C, IH	C, IH	C, IH	C, IH		C, IH	C, IH	C, IH	108, 163–167
*Leishmania sp*.	NR				SC, DEH	C	C	C			C	C	107, 108
New world screwworm	1990				C	C	C	C	C	C	C	C	107, 108
Old world screwworm	NR				C	C	C	C	C	C	C	C	107, 108
*Taenia solium*	Yes	42–59.2%	76, 168, 169	C, IH	C, IH		O, IH			O, IH		C, DH, IH	107, 108
*Trichinella sp*.	Yes	13.3%	170	C	C						SC	C	107, 171, 172
*Trypanosoma evansi*	NR				C	C	O	O		C	C	O	107, 108
*Trypanosoma sp*.	NR				C	C	C	C		U	C	C	108

The table presents the results of the host susceptibility classification for 45 pathogens known to impact swine. In addition, the known status (present/absent) of the pathogens in North American wild pigs along with the reported prevalence range are included. If the pathogen was historically present but has been eradicated from North America the year of eradication is provided and pathogens never reported are indicated as NR. Wild pigs are included specifically to identify gaps in available scientific data for differences in susceptibility between domestic swine and wild pigs. Pathogens noted with ^†^ were not OIE listed at the time of analysis however are included here for completeness and were not included in network analyses.

### Implementation of analytical methods

All statistical and network analyses were implemented in the R computing environment^51^. Network analyses were implemented using the Network Analysis and Visualization (igraph) package^52^. Linear regression and descriptive statistics were calculated using base functions in R. Standard deviations and confidence intervals of proportions were calculated using the score interval approximation method^53^.

## Results

### Identification of shared pathogens

Our assessment using a structured literature review and expert panel identified 39 (46%) of the 84 OIE terrestrial pathogens as those that can affect swine, with 22 (56%) viral, 9 (23%) bacterial, and 8 (21%) parasitic pathogens (Table 2). Of these 39 pathogens affecting swine, 33 (85%) caused clinical disease while only a few (4; 10%) were categorized as causing asymptomatic (or subclinical) infection or had documented natural infections in swine with unknown consequences in (2; 5%). Our assessment of all species’ susceptibility to pathogens of swine found that of these 39 pathogens, 34 (87.2%) caused clinical or sub-clinical disease in at least one other species. On average 70% (±25%; ±StDev) of swine pathogens could infect other species (Table 3). Specifically, non-swine hosts were susceptible (clinical, subclinical, affected, and occasionally affected) to 80% (±32%) of bacterial, 56% (±13%) of viral, and 73% (±24%) of swine parasitic pathogens. All species except for poultry were susceptible to greater than 75% of bacterial pathogens. All species except poultry and cervids were susceptible to more than 75% of parasites; humans had the greatest number, being susceptible to 100% of parasites evaluated. Susceptibility to swine viral pathogens was the lowest among other host species with *Bovidae* (cattle, sheep, goat) being the most susceptible (>60%) to swine viral pathogens (see Table 2). On average 73% (±29%) of bacterial, 39% (±13%) of viral, and 63% (±20%) of swine parasitic pathogens caused clinical disease in other species. All species except poultry and cervids had greater than 75% of swine bacterial pathogens causing clinical disease. Humans accounted for the greatest proportion of swine viral pathogens causing clinical disease (88%) while cattle, humans, and horses accounted for the greatest number of parasitic pathogens causing clinical disease. We also documented studies that specifically investigated wild pigs for susceptibility to domestic swine diseases. Nearly all 8 (80%) of the bacterial diseases had been investigated using wild pigs. Only 10 (37%) of the viral pathogens and 3 (37%) of the parasitic pathogens had been investigated in wild pigs.

**Table 3:** All swine pathogens causing clinical and sub-clinical disease in livestock, poultry, cervids and humans.

	Cattle	Sheep	Goats	Horse	Cervids	Poultry	Humans	Mean	StdDev
**% Shared**									
Bacterial	100	100	100	100	75	12.5	75	80.4	32.2
Viral	75	87.5	75	87.5	62.5	25	100	73.2	24.4
Parasitic	66.7	61.9	71.4	52.4	47.6	33.3	57.1	55.8	12.8
All	75.7	75.7	78.4	70.3	56.8	27	70.3	64.9	18.1
**Eigenvalue Centrality**								**Mean**	**Min/Max**
Bacterial	1	1	1	1	0.80	0.17	0.80	0.82	0.17–1
Viral	0.99	0.98	1	0.88	0.78	0.65	0.92	0.89	0.65–1
Parasitic	0.98	1	0.98	0.97	0.77	0.42	0.99	0.87	0.42–1
All	1	0.99	1	0.92	0.79	0.46	0.90	0.87	0.46–1

### Transmission potential

**Figure 1:**
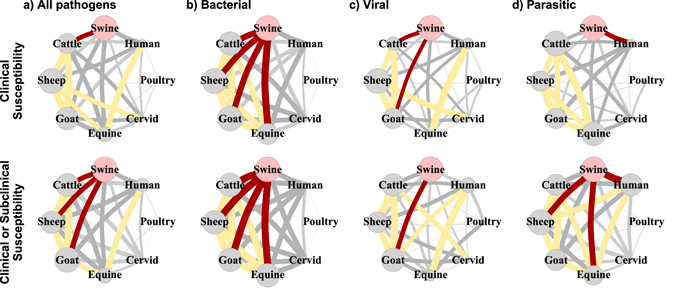
Transmission potential networks used in this study created by connecting two host species if they were susceptible to the same pathogen causing clinical or subclinical disease in swine. Top row are pathogens causing clinical disease in non-swine hosts and the bottom row are all pathogens affecting non-swine hosts. Edge weight between two species is the similarity in the parasites infecting a pair of individuals calculated with the Jaccard index. Red edges denote Jaccard index in the upper 75th quartile, while light gray are edges in the lower 25th quartile. Node size indicates the relative centrality of the species group in the transmission network, calculated using the eigenvalue centrality – more central nodes are larger.

Transmission potential, measured using the Jaccard index, between swine and other species demonstrated heterogeneity. Figure 1 illustrates the transmission potential between swine and other species. Members of the family *Bovidae* were important (upper 75^th^ quartile Jaccard index) for all but parasitic pathogens causing clinical disease. When all pathogens were considered together cattle was the only species group in the upper 75^th^ quartile. Transmission potential between swine and multiple species was greatest for bacterial pathogens with cattle, sheep, goat, and horse all having Jaccard index values in the upper 75^th^ quartile. Viral pathogen transmission with swine was greatest for cattle and goats. In our study parasitic pathogen transmission potential with swine was highest for humans. In networks considering all types of susceptibility cattle, sheep, and goat had the greatest relative transmission potential with swine. There was little difference between bacterial pathogen networks for clinical susceptibility and all susceptibilities. Parasitic transmission potential with swine increased with sheep, horse and humans all in the upper 75^th^ quartile.

Centrality for species demonstrated less heterogeneity (Tables 3 and 4). Cattle, sheep and goat consistently had the greatest centralities (mean EV = 0.99; 0.98–1; min-max) while poultry had lower network centrality (EV = 0.40; 0.17–0.65) across all networks and had the lowest centrality (EV = 0.17) for bacterial pathogens. Human centrality (EV = 0.85; 0.77–0.99) was also low for all but the network considering all potential species susceptibilities to parasitic pathogens, in which it had the largest centrality (EV = 0.99).

**Table 4:** Swine pathogens that cause clinical disease in livestock, poultry, cervids and humans.

	Cattle	Sheep	Goats	Horse	Cervids	Poultry	Humans	Mean	StdDev
**% Shared**									
Bacterial	87.5	87.5	87.5	100	62.5	12.5	75	73.2	29.3
Viral	75	62.5	62.5	75	50	25	87.5	62.5	20.4
Parasitic	42.9	42.9	47.6	42.9	28.6	14.3	52.4	38.8	13
All	59.5	56.8	59.5	62.2	40.5	16.2	64.9	51.4	17.4
**Eigenvalue Centrality**								**Mean**	**Min/Max**
Bacterial	1.00	1.00	1.00	0.98	0.77	0.20	0.77	0.82	0.20–1
Viral	0.91	1.00	0.98	0.83	0.77	0.42	0.88	0.83	0.42–1
Parasitic	1.00	0.98	0.98	1.00	0.77	0.53	0.85	0.87	0.53–1
All	0.98	1.00	0.99	0.93	0.77	0.37	0.84	0.84	0.37–1

Pathogen centrality had greater heterogeneity when compared to species centrality (see Supplemental Table S4). Twenty four (70.6%) pathogens had eigenvector centralities greater than 0.5 and normalized degree centralities greater than 0.5, indicating they could be transmitted to at least half of the species considered. Only nine (26.5%) of the pathogens had centrality values below 0.5. Bacterial pathogens on average had greater centrality (EV = 0.86; 0.58–1; DG = 0.78; 0.5–1) than viral (EV = 0.58; 0.17–0.96; DG = 0.52; 0.13–0.88) and parasitic pathogens (EV = 0.73; 0.30–1; DG = 0.68; 0.26–1). The upper 75^th^ quartile of centralities were composed of three bacterial pathogens (*Bacillus anthracis*, *M*. *tuberculosis*, *B*. *abortus*), three parasitic pathogens (*Chrysomya putoria*, *Cochliomyia hominivorax*, *Echinococcosis sp*.), and one virus (*Lyssavirus sp*.) (Table S2). Pathogens with the smallest centralities were largely viral, with the lower 25^th^ quartile of centralities composed of six viruses (*Equine influenza*, *Asfivirus sp*., *Pestivirus sp*., *Arterivirus sp*., *Enterovirus B*, *Alphacoronavirus 1*), and two parasites (*Trichinella spp*, *Taenia solium*).

### Current status of pathogens in North America

**Figure 2:**
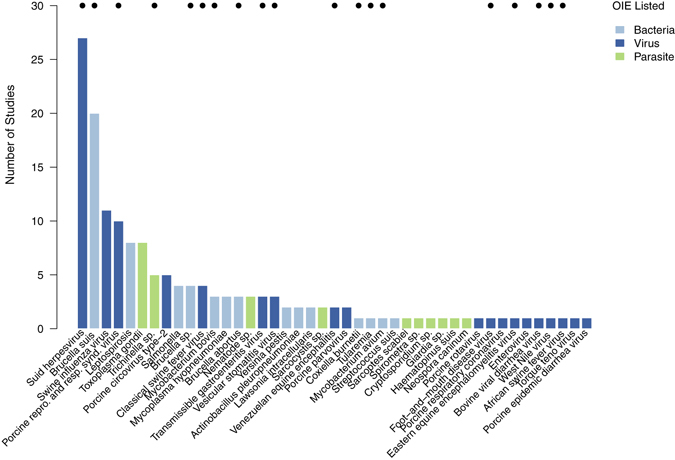
Number of scientific peer reviewed publications (n = 72) reporting results of prevalence studies for wild pigs in North America. Dots along top margin indicate OIE listed swine pathogens (n = 19) of the total number of pathogens (n = 48) with studies.

Sampling of the literature for surveillance studies in North American wild pigs identified 72 publications reporting studies for 48 pathogens. The majority of studies 70 (97%) described surveillance findings from wild pig populations in the United States. We identified one study reporting surveillance results for six pathogens from Sierra La Laguna Biosphere Reserve in Mexico^54^. There was also a single study from the Canadian province of Saskatchewan that reported surveillance results for twelve pathogens^55^. The earliest publication we identified was from 1962 describing epidemiological findings for leptospirosis in wild pigs in Georgia^56^ while the majority (61%) of publications were from the last 20 years. Ten pathogens accounted for 64% of the scientific studies with two, *Brucella suis* and *Suid herpesvirus* (Aujeszky’s disease virus), accounting for 30% of studies (Fig. 2; Table S5). Viral pathogens accounted for the largest number (49%) of surveillance studies while bacterial pathogens accounted for 35%. Thirteen parasites had surveillance studies and *Toxoplasma gondii* accounted for 33% of these studies. Only 49% of OIE listed swine diseases (Table 2) had published surveillance studies reporting findings (positive or negative) in wild pigs and 41% of studies described surveillance results for non-OIE listed pathogens. For pathogens of swine that cause clinical disease in other species 15 (45%) had surveillance studies published. Reported prevalence for these 18 pathogens ranged from 0% to 100%, with vesicular stomatitis virus having the highest reported prevalence (100%) for a single population on Ossobaw island, Georgia^57^.

### Farms and rural populations potentially at risk

**Figure 3:**
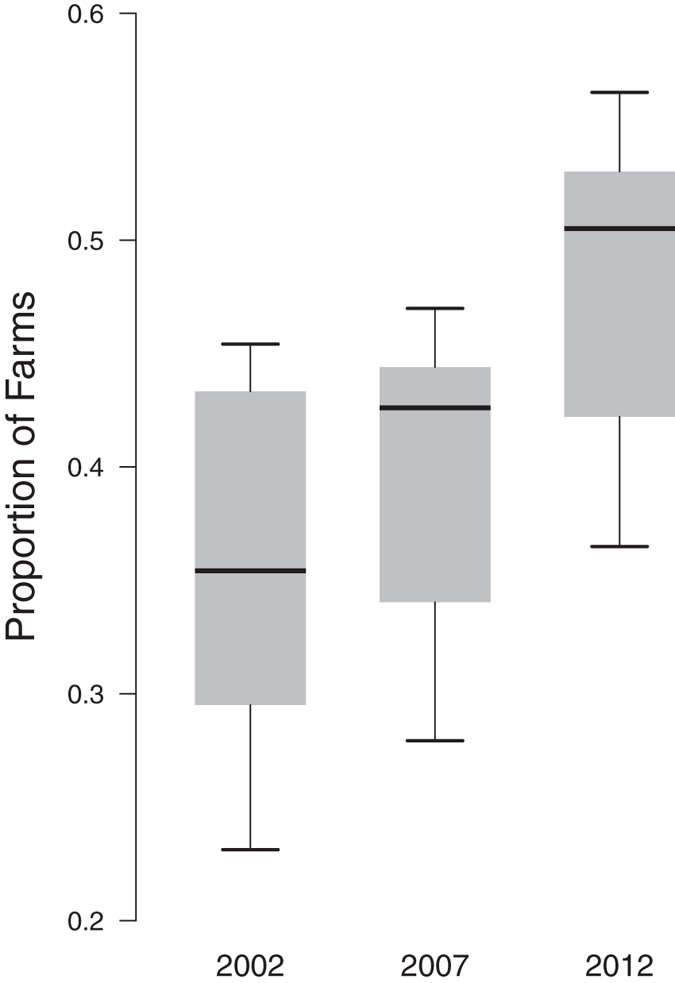
Increase in the proportion of United States farms co-occurring with wild pigs over the ten years we investigated. Boxplots represent the interquartile range (gray box) with the median noted as a solid line, and the whiskers indicate the minimum and maximum of the data.

**Figure 4:**
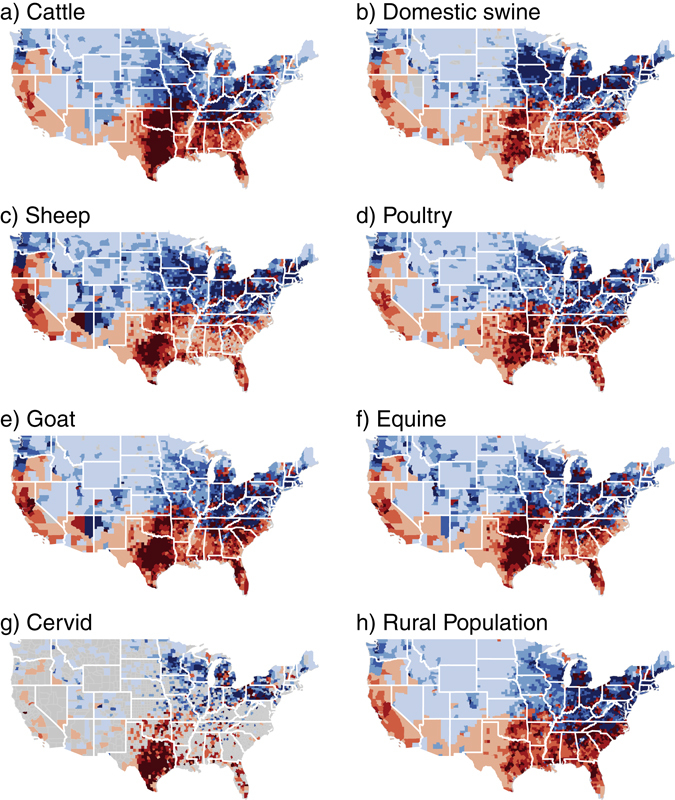
County level co-occurrence of wild pigs, agricultural commodities, and rural human populations in the contiguous United States for 2012. Red shading denotes by quartile the absolute farms density (farms per km^2^) or rural human population density (people per km^2^) within counties co-occurring with wild pigs while blue shading indicates counties without wild pigs. Maps were generated by combining publically available data (see methods) describing wild pig distribution from Southeast Cooperative Wildlife Disease Study (SCWDS), agriculture data from National Agricultural Statistics Service (NASS) Quick Stats database, and rural human population data available from the United States Census Bureau. Maps were created using the maptools package version 0.9.2^101^ in R version 3.3.0^102^.

The co-occurrence of wild pigs and farms for all commodities increased across the ten years investigated (Fig. 3). For the year 2012 on average 47.7% (range 56.5–36.5%) of all farms were in counties with wild pigs representing 46.6% (range 77.3–11.3%) of all domestic animals. The geographic co-occurrence for 2012 is illustrated in Fig. 4 and shows high densities of concordance in the Midwestern states of Texas, Oklahoma, Arkansas, western states of California and Oregon, and eastern states of South Carolina, North Carolina and Florida. Farmed cervids had the largest increase resulting in a 66.6% increase in co-occurrence across the ten years. In 2012, 56.5% of all cervid farms representing 77.3% of all animals were in counties where wild pigs were present. Four of the seven agricultural commodities investigated had over 40% of farms in counties with wild pigs. Domestic swine, an agricultural commodity of concern for disease transmission from wild pigs, had a 58% increase in co-occurrence and an annual rate of increase of 1.3% (95% CI = 1.0–1.7%), with 36.5% farms and 11.3% of animals in counties with wild pigs. Rural human populations had a 29.9% increase in co-occurrence with wild pigs and an annual rate of increase of 1.07% (95% CI = 0.5–1.7%). In 2012 an estimated 46.5% of all rural U.S. citizens lived in counties with wild pigs.

## Discussion

Properties of the transmission potential networks provide an increased understanding of the potential risks of pathogen sharing among species. The majority (87%) of swine pathogens can be transmitted to other species; however this transmission potential was not evenly distributed across species. Both the co-occurrence of wild pigs with family *Bovidae* (cattle, sheep, goat) and the importance of these species in the transmission networks indicate a risk for transmission between *Bovidae* species and wild pigs. *Bovidae* had the highest network metrics indicating greater relative importance among the species and across all swine pathogens. Central nodes are often interpreted in epidemiological networks as being important for network wide transmission^7, 58, 59^, and the same may be true in transmission networks based on pathogen susceptibility^34^. This suggests that the family *Bovidae*, particularly cattle, may be important for transmitting pathogens between swine and other species. Commingling of wild pigs with cattle, sheep and goats is common throughout North America where domestic and wild ruminants share pasture resources^1, 60^. Based on our analysis of wild pig occurrence data, greater than 50% of all U.S. cattle, sheep and goat co-occur in a county with wild pigs. Commingling of livestock, particularly cattle, with wildlife has been associated with the introduction of several pathogens into wildlife populations^61, 62^.

In addition to species heterogeneity, pathogens demonstrated heterogeneity that may be important for transmission among host species. Vector borne pathogens made up less than 23% of pathogens indicating that those pathogens with direct transmission or transmission via fomites may be more likely to cross between species. Yet, despite their low overall frequency, vector borne pathogens were among the highest centralities for viral pathogens. The high potential for cross species transmission and the potential for expanding vector populations due to climate change^63^ highlights the potential risk posed by these pathogens. Vector borne pathogens can be among the most difficult to control once established^64^ and often cause long term challenges for disease risk mitigation.

Excluding vector borne pathogens, fourteen pathogens accounted for 77% of the pathogen network centrality, with greater than two thirds of these being bacterial and parasitic. In the case of bacterial pathogens, *B*. *abortus* and *M*. *bovis* had the highest centrality, when *B*. *anthracis* a pathogen commonly transmitted in the environment, was excluded. These two pathogens have challenged disease control programs in North America for over a century. More recently wild pigs have been established as a maintenance host for *M*. *bovis* in several populations globally^65, 66^ and may pose a risk for transmission in North America^67^. Cross species transmission may be of particular concern in regions with increased commingling of at-risk cattle with wild pigs^60^ and in regions such as the state of Michigan or Riding Mountain National park in Canada where *M*. *bovis* is endemic in wildlife^68^. Broadly our network centrality findings were similar to an inventory of known livestock pathogens that found 77% infect multiple hosts^69^, a study of human pathogens that found 73% are zoonotic^70^, and a study of OIE domestic animal pathogens that found 79% can be transmitted between wildlife and domestic animals^3^.

Non-vector borne viral pathogens with the largest transmission potential between wild pigs and other species included avian influenza virus, foot-and-mouth disease virus and *Suid herpesvirus 1*. Interestingly for some pathogens, particularly avian influenza virus, there was a high transmission potential in the pathogen networks (see Table S4 in supplemental material) and a relatively low transmission potential for the primary host (poultry) in the host networks. There are several potential explanations for this apparent incongruity. Avian influenza viruses’ natural host is wild waterfowl that were not included in our networks. This may indicate that other wildlife species such as waterfowl may have important connections across a diversity of hosts. More importantly this incongruity may indicate that some pathogens have a greater risk for cross species transmission despite low host connectance.

Pathogens, such as avian influenza virus and many of the bacterial pathogens have large host ranges often being able to adapt relatively quickly to new hosts and our approach highlights this characteristic. Our results also clarify which pathogens might be of greater concern requiring additional surveillance. For example the recent emergence of highly pathogenic avian influenza in North America^71^, the potential for swine (domestic or wild) to influence antigenic changes in the virus^72^, and serologic evidence of wild pigs being exposed to influenza^27, 73^, highlights the potential importance of influenza surveillance in domestic and wild pigs. In North America, wild pigs have been documented as actively infected with and having contributed to the transmission of only a fraction of the pathogens we investigated and their contribution to the persistence of these pathogens is still largely uncharacterized^9^. Given the large number of swine pathogens we found that might be transmitted among species, the potential for wild pigs to become an unmonitored reservoir for many pathogens is a concern requiring further inquiry and monitoring.

Despite effort to establish prevalence estimates for wild pigs (see supplemental Table S5), there are gaps for pathogens of interest for human, wildlife, and livestock health. We found discordance between the available surveillance studies and the pathogens that can be shared across species. More than 50% of pathogens that cause clinical disease in other species did not have any North American studies of prevalence in wild pigs. This contrasts with the potential exposure of livestock to wild pigs; domestic animals such as cattle and sheep, that are largely pasture raised in North America, have a potential for coming into contact (directly or indirectly) with wild pigs^74, 75^ and share nearly 90% (see Table 4) of swine pathogens causing clinical infection. Those studies that do report prevalence are generally limited to local or regional investigations^76, 77^. While providing important data, local studies may not represent regional or national prevalence. We found only a few studies^20, 78^ that report prevalence and epidemiological patterns of infection at national or near national scales. Pathogens that did have multiple studies in different regions (e.g. leptospirosis, pseudorabies virus, swine brucellosis, and bovine tuberculosis) had prevalence estimates that ranged from 0–87% indicating spatial heterogeneity in prevalence and transmission risks likely occur. This result may be complicated by true and false detection errors that few studies addressed when reporting findings^79^ and can have large effects on estimated disease prevalence in wildlife^80^. Comprehensive surveillance systems that integrate livestock, wildlife, and human components have been previously identified as a need^81^. Explicitly accounting for the transmission potential and historic geospatial distribution of pathogens to prioritize surveillance (both livestock and wildlife) may offer benefits and reduce knowledge gaps for pathogens of concern for human, wildlife, and livestock health^82^. Developing a comprehensive national monitoring system that integrates domestic and wild animal surveillance, prioritizes pathogens based on transmission risk, potential consequences, and knowledge of occurrence could yield economic benefits for livestock health by reducing spillover events through early detection and risk mitigation^83, 84^.

Incomplete knowledge of the presence of pathogens in wild pig populations and the transmission potential we found may pose risks for foreign animal diseases in North America where wild pigs are potential hosts. The potential economic impacts resulting from disease outbreaks that include wildlife can be large^18, 85–87^ and have long lasting effects on economies and production systems^86, 88^. Livestock production in the United States, that is increasingly interconnected and concentrated^89, 90^, is also becoming more globally reliant^91, 92^. The importance of exports in sustaining market opportunities for United States agriculture has increased, with over 20% of production value exported in 2012^93^. As a result, disease threats to food safety or livestock health that may originate in wildlife have the potential to impact economies^4, 94, 95^. Despite potential economic impacts, assessments that explicitly link disease outbreaks involving wildlife and livestock with changes in export value are currently unavailable. Methods that link disease risk at the wildlife-livestock interface and compare the benefits and costs of risk management (e.g. surveillance, bio-security, etc) in both livestock, wildlife have been proposed^3, 96, 97^, however they have not been extended to risk management at a macro-economic scale.

Further, the expansion of wild pigs has resulted in a large portion of agriculture production and human populations occurring in regions where wild pigs are present (Fig. 4). For the livestock commodities we investigated all had large proportions of farms in regions with wild pigs and none had declines in co-occurrence with wild pig populations. This large proportion of overlap of agricultural and rural populations is increasing as wild pig populations expand in North America^98^. Pathogen exposure risk to both agriculture and humans, along with the potential economic impacts^99^, highlight the need for quantitative analysis and consequence assessments of the risks wild pigs pose to agriculture and human health^3^. Recent analysis by Tompkins, *et al*.^100^ found that disease emergence at the wildlife-livestock interface is often driven by human-induced activities and exposure to domestic animals. Further, Jones, *et al*.^2^ estimated that the rate of future zoonotic disease emergence/reemergence will be closely linked to changes in the agricultural-wildlife nexus. Several studies^2, 3, 100^ have also found that available research and tools inadequately addresses these complex problems limiting prediction, prevention, and mitigation. Given the findings of these studies together with ours, it would be broadly useful to develop approaches for the wildlife-livestock interface that link risk assessments and economic consequence assessments allowing evaluation of the relative benefits and costs of surveillance and risk mitigation, not only for invasive wild pigs, but for a diversity of wildlife-agricultural disease conflicts.

Our transmission networks highlight the potential for cross species transmission between wild pigs, livestock, cervids, and humans. They also highlight heterogeneity in both species and pathogens indicating some species are more important and that some pathogens maybe more frequently transmitted. Additional work is needed to establish the risk of exposure and transmission for pathogens of concern to humans and livestock and may necessitate surveillance studies elucidating potential risks for pathogens of greatest transmission potential. While a complete picture of the risks of wild pig associated diseases is not currently possible, the risk assessment process is valuable for prioritizing knowledge gaps. Evaluation of potential, but unstudied, impact of wild pigs on the consequences of disease (e.g. outbreak duration, extent, effectiveness of disease management) maybe warranted. As the first comprehensive assessment of cross-species diseases associated with wild pigs, these results are an early step to characterize and prioritize the disease risks as wild pig populations expand.

## Electronic supplementary material

Supplementary Information

Supplementary Dataset 1

Supplementary Dataset 2

Supplementary Dataset 3

## References

[1] Miller RS, Sweeney SJ (2013). Mycobacterium bovis (bovine tuberculosis) infection in north american wildlife: Current status and opportunities for mitigation of risks of further infection in wildlife populations. Epidemiology and infection.

[2] Jones BA (2013). Zoonosis emergence linked to agricultural intensification and environmental change. Proceedings of the National Academy of Sciences of the United States of America.

[3] Miller RS, Farnsworth ML, Malmberg JL (2013). Diseases at the livestock–wildlife interface: Status, challenges, and opportunities in the united states. Preventive veterinary medicine.

[4] Jones KE (2008). Global trends in emerging infectious diseases. Nature.

[5] Taylor LH, Latham SM, Mark E (2001). Risk factors for human disease emergence. Philosophical Transactions of the Royal Society of London B: Biological Sciences.

[6] Siembieda J, Kock R, McCracken T, Newman S (2011). The role of wildlife in transboundary animal diseases. Animal Health Research Reviews.

[7] Luis AD (2015). Network analysis of host–virus communities in bats and rodents reveals determinants of cross‐species transmission. Ecology letters.

[8] Lloyd-Smith JO (2009). Epidemic dynamics at the human-animal interface. Science.

[9] Bevins SN (2014). Consequences associated with the recent range expansion of nonnative feral swine. BioScience.

[10] U.S. Department of Agriculture. *Final environmental impact statement, feral swine damage management: A national approach* 550 (2015).

[11] Barrios-Garcia MN, Ballari SA (2012). Impact of wild boar (sus scrofa) in its introduced and native range: A review. Biological Invasions.

[12] Brook RK, van Beest FM (2014). Feral wild boar distribution and perceptions of risk on the central canadian prairies. Wildlife Society Bulletin.

[13] McClure ML (2015). Modeling and mapping the probability of occurrence of invasive wild pigs across the contiguous united states. PloS one.

[14] Müller T (2011). Pseudorabies virus in wild swine: A global perspective. Archives of virology.

[15] Jori F, Bastos AD (2009). Role of wild suids in the epidemiology of african swine fever. EcoHealth.

[16] Reiner G, Fresen C, Bronnert S, Willems H (2009). Porcine reproductive and respiratory syndrome virus (prrsv) infection in wild boars. Veterinary microbiology.

[17] Baldock, C. *et al*. New technologies in the fight against transboundary animal diseases. Report No. 9251043582, (Food and Agriculture Organizations of the United Nations, Rome, Italy, 1999).

[18] Cozzens, T. W. *Economic impact of feral swine transmitting foot-and-mouth disease to livestock in kansas* Master of Science thesis, Colorado State University, (2010).

[19] Cozzens, T. *et al*. In *Proceedings 24th Vertebrate* Pest *Confrence* (eds R. M. Timm & K. A. Fagerstone) 308–311 (University of California Davis, 2010).

[20] Pedersen K (2012). Apparent prevalence of swine brucellosis in feral swine in the united states. Human-Wildlife Interactions.

[21] Boadella M, Gortázar C, Vicente J, Ruiz-Fons F (2012). Wild boar: An increasing concern for aujeszky’s disease control in pigs?. BMC veterinary research.

[22] Torre A (2015). Assessing the risk of african swine fever introduction into the european union by wild boar. Transboundary and emerging diseases.

[23] Coffey, B. *et al*. *The economic impact of bse on the us beef industry: Product value losses, regulatory costs, and consumer reactions* 68 (Kansas State University, Manhattan, Kansas, 2005).

[24] Li T-C (2005). Hepatitis e virus transmission from wild boar meat. Emerging Infectious Diseases.

[25] Holzbauer SM (2014). Outbreak of trichinella spiralis infections associated with a wild boar hunted at a game farm in iowa. Clinical Infectious Diseases.

[26] Rodríguez DLPE (2004). Trichinellosis outbreaks in spain (1990–2001). Enfermedades infecciosas y microbiología clínica.

[27] Feng Z (2014). Influenza a subtype h3 viruses in feral swine, united states, 2011–2012. Emerging infectious diseases.

[28] Hamano M (2007). Detection of antibodies to japanese encephalitis virus in the wild boars in hiroshima prefecture, japan. Epidemiology and infection.

[29] Jay MT (2007). Escherichia coli o157: H7 in feral swine near spinach fields and cattle, central california coast. Emerging infectious diseases.

[30] Jay‐Russell M (2012). Isolation of campylobacter from feral swine (sus scrofa) on the ranch associated with the 2006 escherichia coli o157: H7 spinach outbreak investigation in california. Zoonoses and public health.

[31] Wiethoelter AK, Beltrán-Alcrudo D, Kock R, Mor SM (2015). Global trends in infectious diseases at the wildlife–livestock interface. Proceedings of the National Academy of Sciences.

[32] World Organisation for Animal Health (OIE). Working group on risk analysis., (World Organisation for Animal Health Regional Commission for the Americas., Paris, France, 1999).

[33] Wieland B, Dhollander S, Salman M, Koenen F (2011). Qualitative risk assessment in a data-scarce environment: A model to assess the impact of control measures on spread of african swine fever. Preventive Veterinary Medicine.

[34] Pilosof S, Morand S, Krasnov BR, Nunn CL (2015). Potential parasite transmission in multi-host networks based on parasite sharing. PloS one.

[35] World Organisation for Animal Health (OIE). Terrestrial animal health code. 435 (World Organisation for Animal Health, Paris, France, 2013).

[36] VanderWaal KL, Atwill ER, Isbell L, McCowan B (2014). Linking social and pathogen transmission networks using microbial genetics in giraffe (giraffa camelopardalis). Journal of animal ecology.

[37] VanderWaal KL, Atwill ER, Isbell LA, McCowan B (2014). Quantifying microbe transmission networks for wild and domestic ungulates in kenya. Biological Conservation.

[38] Koleff P, Gaston KJ, Lennon JJ (2003). Measuring beta diversity for presence–absence data. Journal of Animal Ecology.

[39] Newman, M. *Networks: An introduction* (Oxford University Press, 2010).

[40] Griffin RH, Nunn CL (2012). Community structure and the spread of infectious disease in primate social networks. Evolutionary Ecology.

[41] Canright GS, Engø-Monsen K (2006). Spreading on networks: A topographic view. Complexus.

[42] Everett MG, Borgatti SP (2005). Extending centrality. Models and methods in social network analysis.

[43] Moher, D. *et al*. Preferred reporting items for systematic reviews and meta-analyses: The prisma statement. *PLoS Med* **6**, doi:10.1371/journal.pmed.1000097 (2009). 10.1371/journal.pmed.1000097 PMC2707599 19621072

[44] Liberati A (2009). The prisma statement for reporting systematic reviews and meta-analyses of studies that evaluate health care interventions: Explanation and elaboration. PLoS Med.

[45] Khan KS, Kunz R, Kleijnen J, Antes G (2003). Five steps to conducting a systematic review. Journal of the Royal Society of Medicine.

[46] Okoli, C. A critical realist guide to developing theory with systematic literature reviews. *Social Science Research Network*, doi:10.2139/ssrn.2115818 (2012).

[47] U.S. Department of Agriculture. *Quick stats*, http://quickstats.nass.usda.gov/ Accessed: April 20, 2014 (2014).

[48] U.S. Census Bureau. *Census 2010 summary file 1 table p2*., http://factfinder2.census.gov Accessed: January 15, 2016 (2010).

[49] Southeastern Cooperative Wildlife Disease Study (SCWDS). *National feral swine distribution maps* (Southeastern Cooperative Wildlife Disease Study, University of Georgia, Athens, GA, 2013).

[50] Cromartie, J. Population and migration (2015).

[51] R: A language and environment for statistical computing v. 3.3.0 (*R Foundation for Statistical Computing*, Vienna, Austria, 2016).

[52] Csardi G, Nepusz T (2006). The igraph software package for complex network research. InterJournal, Complex Systems.

[53] Agresti, A. & Kateri, M. *Categorical data analysis* (Springer, 2011).

[54] Rivera, C. M. P., López, M. S., Franco, G. A. & Nápoles, R. C. Detection of antibodies against pathogens in feral and domestic pigs (sus scrofa) at the sierra la laguna biosphere reserve, mexico. *Veterinaria México OA* **4** (2017).

[55] McGregor GF (2015). Disease risks associated with free-ranging wild boar in saskatchewan. The Canadian Veterinary Journal.

[56] Gorham GW, McKeever S, Grimes RD, Gorman GW (1962). Leptospirosis in wild mammals from southwestern georgia. American journal of tropical medicine and hygiene.

[57] Stallknecht D, Nettles V, Erickson G, Jessup D (1986). Antibodies to vesicular stomatitis virus in populations of feral swine in the united states. Journal of wildlife diseases.

[58] Craft ME, Caillaud D (2011). Network models: An underutilized tool in wildlife epidemiology?. Interdisciplinary perspectives on infectious diseases.

[59] Paull SH (2012). From superspreaders to disease hotspots: Linking transmission across hosts and space. Frontiers in Ecology and the Environment.

[60] Cooper SM (2010). Distribution and interspecies contact of feral swine and cattle on rangeland in south texas: Implications for disease transmission. Journal of Wildlife Diseases.

[61] Cross PC (2007). Effects of management and climate on elk brucellosis in the greater yellowstone ecosystem. Ecological Applications.

[62] Maichak EJ (2009). Effects of management, behavior, and scavenging on risk of brucellosis transmission in elk of western wyoming. Journal of Wildlife Diseases.

[63] Rochlin I, Ninivaggi DV, Hutchinson ML, Farajollahi A (2013). Climate change and range expansion of the asian tiger mosquito (aedes albopictus) in northeastern USA: Implications for public health practitioners. PloS one.

[64] Gubler DJ (1998). Resurgent vector-borne diseases as a global health problem. Emerging infectious diseases.

[65] Aranaz A (2004). Bovine tuberculosis (mycobacterium bovis) in wildlife in spain. Journal of Clinical Microbiology.

[66] Santos N (2009). Epidemiology of mycobacterium bovis infection in wild boar (sus scrofa) from portugal. Journal of Wildlife Diseases.

[67] Pedersen K (2016). Limited antibody evidence of exposure to mycobacterium bovis in feral swine (sus scrofa) in the USA. Journal of Wildlife Diseases.

[68] Ramsey DS (2014). Forecasting eradication of bovine tuberculosis in michigan white‐tailed deer. The Journal of Wildlife Management.

[69] Cleaveland S, Laurenson M, Taylor L (2001). Diseases of humans and their domestic mammals: Pathogen characteristics, host range and the risk of emergence. Philosophical Transactions of the Royal Society of London B: Biological Sciences.

[70] Woolhouse, M. E. & Gowtage-Sequeria, S. *Host range and emerging and reemerging pathogens* Vol. 192 306 (The National Academies Press, 2006). 10.3201/eid1112.050997 PMC3367654 16485468

[71] Bevins SN (2016). Widespread detection of highly pathogenic h5 influenza viruses in wild birds from the pacific flyway of the united states. Scientific Reports.

[72] Kuntz‐Simon G, Madec F (2009). Genetic and antigenic evolution of swine influenza viruses in europe and evaluation of their zoonotic potential. Zoonoses and public health.

[73] Hall JS (2008). Influenza exposure in united states feral swine populations. Journal of wildlife diseases.

[74] Barasona JA (2014). Spatiotemporal interactions between wild boar and cattle: Implications for cross-species disease transmission. Veterinary research.

[75] Cowie CE (2016). Interactions between four species in a complex wildlife: Livestock disease community: Implications for mycobacterium bovis maintenance and transmission. European journal of wildlife research.

[76] Corn JL, Cumbee JC, Barfoot R, Erickson GA (2009). Pathogen exposure in feral swine populations geographically associated with high densities of transitional swine premises and commercial swine production. Journal of wildlife diseases.

[77] Van der Leek M (1993). Prevalence of pseudorabies (aujeszky’s disease) virus antibodies in feral swine in florida. Journal of wildlife diseases.

[78] Pedersen K (2013). Pseudorabies in feral swine in the united states, 2009–2012. Journal of wildlife diseases.

[79] McClintock BT (2010). Seeking a second opinion: Uncertainty in disease ecology. Ecology letters.

[80] Jennelle CS, Cooch EG, Conroy MJ, Senar JC (2007). State-specific detection probabilities and disease prevalence. Ecological Applications.

[81] Stallknecht, D. In *Wildlife and emerging zoonotic diseases: The biology*, *circumstances and consequences of cross-species transmission Current topics in microbiology and immunology* (eds James E. Childs, John S. Mackenzie, & Jürgen A. Richt) 445–461 (Springer Berlin Heidelberg, 2007).

[82] McKenzie J, Simpson H, Langstaff I (2007). Development of methodology to prioritise wildlife pathogens for surveillance. Preventive veterinary medicine.

[83] Jebara KB (2004). Surveillance, detection and response: Managing emerging diseases at national and international levels. Rev Sci Tech.

[84] Wendt A, Kreienbrock L, Campe A (2015). Zoonotic disease surveillance–inventory of systems integrating human and animal disease information. Zoonoses and public health.

[85] Anderson, D. *et al*. Economic impact of expanded fever tick range. *Agricultural & Food Policy Center*, *Texas A&M University*, *College Station*, *TX* (2010).

[86] Epstein JH (2006). Nipah virus: Impact, origins, and causes of emergence. Current Infectious Disease Reports.

[87] O’Brien DJ, Schmitt SM, Fitzgerald SD, Berry DE (2011). Management of bovine tuberculosis in michigan wildlife: Current status and near term prospects. Veterinary microbiology.

[88] Knight-Jones T, Rushton J (2013). The economic impacts of foot and mouth disease–what are they, how big are they and where do they occur?. Preventive veterinary medicine.

[89] Reimer JJ (2006). Vertical integration in the pork industry. American journal of agricultural economics.

[90] Martinez, S. Vertical coordination of marketing systems: Lessons from the poultry, egg and pork industries. Report No. AER-807, 45 (United States of America, 2012).

[91] McCullough, E. B., Pingali, P. L. & Stamoulis, K. G. *The transformation of agri-food systems: Globalization*, *supply chains and smallholder farmers* (Food and Agriculture Organization of the United Nations and Earthscan, 2008).

[92] Bonanno, A. *From columbus to conagra: The globalization of agriculture and food* (University Press of Kansas, 1994).

[93] Jerardo, A. *Export share of production*, https://www.ers.usda.gov/topics/international-markets-trade/us-agricultural-trade/export-share-of-production/#Production January 26, 2017 (2012).

[94] Fidler DP (1996). Globalization, international law, and emerging infectious diseases. Emerging infectious diseases.

[95] Daszak P, Cunningham AA, Hyatt AD (2000). Emerging infectious diseases of wildlife–threats to biodiversity and human health. Science.

[96] Shwiff SA (2016). A benefit-cost analysis decision framework for mitigation of disease transmission at the wildlife–livestock interface. Human-Wildlife Interactions.

[97] Horan RD, Fenichel EP (2007). Economics and ecology of managing emerging infectious animal diseases. American journal of agricultural economics.

[98] Snow NP, Jarzyna MA, VerCauteren KC (2017). All the way home: Interpreting and predicting the spread of invasive wild pigs. Journal of Applied Ecology.

[99] Anderson A (2016). Economic estimates of feral swine damage and control in 11 us states. Crop Protection.

[100] Tompkins DM (2015). Emerging infectious diseases of wildlife: A critical perspective. Trends in parasitology.

[101] Bivand, R. & Lewin-Koh, N. Vol. R package version 0.9.2 (2016).

[102] R Core Team. Vol. 3.3.0 (*R Foundation for Statistical Computing*, Vienna, Austria, 2014).

[103] Turnbull P (1992). Serology and anthrax in humans, livestock and etosha national park wildlife. Epidemiology and infection.

[104] Williams, E. S. & Barker, I. K. *Infectious diseases of wild mammals* 3 edn, 576 (John Wiley & Sons, 2008).

[105] Zimmerman, J. J. *et al*. *Diseases of swine* 10 edn, (Wiley-Blackwell, 2012).

[106] Spickler, A. R. *Anthrax*, http://www.cfsph.iastate.edu/DiseaseInfo/factsheets.php April 2, 2016 (2007).

[107] Coetzer, J., Thomson, G. & Tustin, R. *Infectious diseases of livestock* Vol. 3 (Oxford University Press Southern Africa, 2004).

[108] Spickler, A. R. *Emerging and exotic diseases of animals* (The Center for Food Security and Public Health, Iowa State University, 2010).

[109] Stoffregen WC (2007). Diagnostic characterization of a feral swine herd enzootically infected with brucella. Journal of veterinary diagnostic investigation.

[110] Spickler, A. R. *Bovine brucellosis: Brucella abortus*, http://www.cfsph.iastate.edu/DiseaseInfo/factsheets.php April 2, 2016 (2009).

[111] Drew ML, Jessup DA, Burr AA, Franti C (1992). Serologic survey for brucellosis in feral swine, wild ruminants, and black bear of california, 1977 to 1989. Journal of wildlife diseases.

[112] Van Der Leek M (1993). Prevalence of brucella sp. Antibodies in feral swine in florida. Journal of wildlife diseases.

[113] Gresham CS (2002). Increased prevalence of brucella suis and pseudorabies virus antibodies in adults of an isolated feral swine population in coastal south carolina. Journal of wildlife diseases.

[114] Zygmont S (1982). Brucellosis in wild swine: A serologic and bacteriologic survey in the southeastern united states and hawaii. Journal of the American Veterinary Medical Association.

[115] Randhawa A, Kelly V, Baker E (1977). Agglutinins to coxiella burnetii and brucella spp, with particular reference to brucella canis, in wild animals of southern texas. Journal of the American Veterinary Medical Association.

[116] Dardiri A, Logan L, Mebus C (1987). Susceptibility of white-tailed deer to experimental heartwater infections. Journal of Wildlife Diseases.

[117] Hartin, R. E., Ryan, M. R. & Campbell, T. A. Distribution and disease prevalence of feral hogs in missouri. **1**, 186–191 (2007).

[118] Morner T (1992). The ecology of tularaemia. Rev Sci Tech.

[119] New JC (1994). A serologic survey of selected viral and bacterial diseases of european wild hogs, great smoky mountains national park, USA. Journal of wildlife diseases.

[120] Chatfield J (2013). Serosurvey of leptospirosis in feral hogs (sus scrofa) in florida. Journal of Zoo and Wildlife Medicine.

[121] Spickler, A. R. *Leptospirosis*, http://www.cfsph.iastate.edu/DiseaseInfo/factsheets.php April 2, 2016 (2013).

[122] Essey, M., Payne, R., Himes, E. & Luchsinger, D. In *Proceedings of the United States Animal Health Association*. 538–549 (1981).

[123] Smith, P. *Bovine-type tuberculosis infection in feral swine* Master of Science thesis, University of California, (1968).

[124] Spickler, A. R. *Bovine tuberculosis*, http://www.cfsph.iastate.edu/DiseaseInfo/factsheets.php April 2, 2016 (2009).

[125] Spickler, A. R. *Hemorrhagic septicemia*, http://www.cfsph.iastate.edu/DiseaseInfo/factsheets.php April 2, 2016 (2009).

[126] Duncan C (2008). Persistent bovine viral diarrhea virus infection in wild cervids of colorado. Journal of veterinary diagnostic investigation.

[127] Passler T (2007). Experimental persistent infection with bovine viral diarrhea virus in white-tailed deer. Veterinary microbiology.

[128] Nettles VF, Corn JL, Erickson GA, Jessup DA (1989). A survey of wild swine in the united states for evidence of hog cholera. Journal of wildlife diseases.

[129] Brody JA, Murray WA (1959). Arthropod-borne encephalitis in the united states, 1957. Public health reports.

[130] Elvinger F (1996). Prevalence of exposure to eastern equine encephalomyelitis virus in domestic and feral swine in georgia. J Vet Diagn Invest.

[131] Schmitt SM (2007). An outbreak of eastern equine encephalitis virus in free-ranging white-tailed deer in michigan. Journal of wildlife diseases.

[132] Tate CM (2005). Eastern equine encephalitis in a free-ranging white-tailed deer (odocoileus virginianus). Journal of wildlife diseases.

[133] Spickler, A. R. *Eastern equine encephalomyelitis*, *western equine encephalomyelitis and venezuelan equine encephalomyelitis*, http://www.cfsph.iastate.edu/DiseaseInfo/factsheets.php April 2, 2016 (2008).

[134] Ruder MG (2012). Susceptibility of white-tailed deer (odocoileus virginianus) to experimental infection with epizootic hemorrhagic disease virus serotype 7. Journal of wildlife diseases.

[135] Breard E (2013). Epizootic hemorrhagic disease virus serotype 6 experimentation on adult cattle. Research in veterinary science.

[136] Guo Y (1995). Seroepidemiological and molecular evidence for the presence of two h3n8 equine influenza viruses in china in 1993–94. Journal of general virology.

[137] Cook RA (2005). Emerging diseases at the interface of people, domestic animals and wildlife. The role of wildlife in our understanding of highly pathogenic avian influenza. The Yale journal of biology and medicine.

[138] Kalthoff D (2008). Experimental infection of cattle with highly pathogenic avian influenza virus (h5n1). Emerging infectious diseases.

[139] Lipatov AS (2008). Domestic pigs have low susceptibility to h5n1 highly pathogenic avian influenza viruses. PLoS pathogens.

[140] Olsen B (2006). Global patterns of influenza a virus in wild birds. Science.

[141] Guo Y (1992). Characterization of a new avian-like influenza a virus from horses in china. Virology.

[142] Morens DM, Taubenberger JK (2010). An avian outbreak associated with panzootic equine influenza in 1872: An early example of highly pathogenic avian influenza?. Influenza and other respiratory viruses.

[143] Spickler, A. R. *Influenza* http://www.cfsph.iastate.edu/DiseaseInfo/factsheets.php April 2, 2016 (2014).

[144] Kumar R (1999). Viral encephalitis of public health significance in india: Current status. The Indian Journal of Pediatrics.

[145] Emord D, Morris C (1984). Epizootiology of eastern equine encephalomyelitis virus in upstate new york, USA vi. Antibody prevalence in wild birds during an interepizootic period. Journal of medical entomology.

[146] Aitken, I. *Diseases of sheep* 4 edn, (Blackwell Publishing, 2008).

[147] Stevenson GW (2013). Emergence of porcine epidemic diarrhea virus in the united states: Clinical signs, lesions, and viral genomic sequences. Journal of veterinary diagnostic investigation.

[148] Saliki JT, Rodgers SJ, Eskew G (1998). Serosurvey of selected viral and bacterial diseases in wild swine from oklahoma. Journal of Wildlife Diseases.

[149] Wyckoff AC (2009). Feral swine contact with domestic swine: A serologic survey and assessment of potential for disease transmission. Journal of Wildlife Diseases.

[150] Scott GR (1963). Pigs and rift valley fever. Nature.

[151] Barrett T, Rossiter P (1999). Rinderpest: The disease and its impact on humans and animals. Advances in Virus Research.

[152] Rossiter, P., Williams, E. S., Munson, L. & Kennedy, S. Morbilliviral diseases. *Infectious Diseases of Wild Mammals*, *Third Edition* 37–76 (2001).

[153] Corn JL (2004). Persistence of pseudorabies virus in feral swine populations. Journal of wildlife diseases.

[154] Woods R, Pirtle E, Sacks J, Gibbs E (1990). Serologic survey for transmissible gastroenteritis virus neutralizing antibodies in selected feral and domestic swine sera in the southern united states. Journal of wildlife diseases.

[155] Stallknecht D, Nettles V, Fletcher W, Erickson G (1985). Enzootic vesicular stomatitis new jersey type in an insular feral swine population. American journal of epidemiology.

[156] Stallknecht DE (1993). Feral swine as a potential amplifying host for vesicular stomatitis virus new jersey serotype on ossabaw island, georgia. Journal of wildlife diseases.

[157] Webb PA (1987). Epizootic vesicular stomatitis in colorado, 1982: Some observations on the possible role of wildlife populations in an enzootic maintenance cycle. Journal of wildlife diseases.

[158] Gibbs SE (2006). Antibodies to west nile virus in feral swine from florida, georgia, and texas, USA. Vector-Borne & Zoonotic Diseases.

[159] Kramer LD, Li J, Shi P-Y (2007). West nile virus. The Lancet Neurology.

[160] Van der Meulen K, Pensaert M, Nauwynck H (2005). West nile virus in the vertebrate world. Archives of virology.

[161] Nemeth NM, Bowen RA (2007). Dynamics of passive immunity to west nile virus in domestic chickens (gallus gallus domesticus). The American journal of tropical medicine and hygiene.

[162] Miller DL, Radi ZA, Baldwin C, Ingram D (2005). Fatal west nile virus infection in a white-tailed deer (odocoileus virginianus). Journal of wildlife diseases.

[163] Leiby PD, Carney WP, Woods CE (1970). Studies on sylvatic echinococcosis. Iii. Host occurrence and geographic distribution of echinococcus multilocularis in the north central united states. The Journal of parasitology.

[164] Storandt S (2002). Distribution and prevalence of echinococcus multilocularis in wild predators in nebraska, kansas, and wyoming. Journal of Parasitology.

[165] Storandt ST, Kazacos KR (1993). Echinococcus multilocularis identified in indiana, ohio, and east-central illinois. The Journal of parasitology.

[166] Thompson R (2006). Molecular and morphological characterization of echinococcus in cervids from north america. Parasitology.

[167] Spickler, A. R. *Echinococcosis* http://www.cfsph.iastate.edu/DiseaseInfo/factsheets.php April 2, 2016 (2011).

[168] Baker, S. R., O’Neil, K. M., Dee, S. A. & Gramer, M. R. Estimates of the seroprevalence of production-limiting diseases in wild pigs. *Veterinary record* **168**, doi:10.1136/vr.d1020 (2011). 10.1136/vr.d1020 21610003

[169] Sandfoss MR (2012). A serosurvey for brucella suis, classical swine fever virus, porcine circovirus type 2, and pseudorabies virus in feral swine (sus scrofa) of eastern north carolina. Journal of wildlife diseases.

[170] Sandfoss M (2011). Prevalence of antibody to toxoplasma gondii and trichinella spp. In feral pigs (sus scrofa) of eastern north carolina. Journal of wildlife diseases.

[171] Murrell K (1987). Trichinella spiralis in an agricultural ecosystem. Ii. Evidence for natural transmission of trichinella spiralis spiralis from domestic swine to wildlife. The Journal of parasitology.

[172] Gajadhar AA, Bisaillon J-R, Appleyard GD (1997). Status of trichinella spiralis in domestic swine and wild boar in canada. Canadian Journal of Veterinary Research.

