# Supplementary material for: Cross-species transmission potential between wild pigs, livestock, poultry, wildlife, and humans: implications for disease risk management in North America
Source: Sci Rep. 2017 Aug 10;7:7821. doi: 10.1038/s41598-017-07336-z (PMC5552697; doi:10.1038/s41598-017-07336-z)
Supplement: Supplementary file 1 — Supplementary Information [file 41598_2017_7336_MOESM1_ESM.pdf]

Supplementary Information

Cross-species transmission potential between wild pigs, livestock, poultry, wildlife, and humans: implications for disease risk management in North America

Ryan S. Miller[1]*, Steven J. Sweeney[1], Chris Slootmaker[2], Daniel A. Grear[3],

Paul A. Di Salvo[1], Deborah Kiser[1], Stephanie A. Shwiff[2]

Authors' Affiliation:

[1] Center for Epidemiology and Animal Health, Veterinary Services, Animal and Plant Health Inspection Service, United States Department of Agriculture, Fort Collins, Colorado

[2] National Wildlife Research Center, Wildlife Services, Animal and Plant Health Inspection Service, United States Department of Agriculture, Fort Collins, Colorado

[3] National Wildlife Health Center, United States Geological Survey, Madison, Wisconsin

*** Correspondence:**

Ryan S. Miller

2150 Centre Avenue, Bldg B

Fort Collins, Colorado, 80526-8117 USA

Email: Ryan.Miller@rsmiller.net; Ryan.S.Miller@aphis.usda.gov

SUPPLEMENTARY INFORMATION

Description of method used to assess and identify shared pathogens

Literature review used to assess species susceptibility

To evaluate the 84 OIE pathogens ability to infect swine (wild and domestic), cattle, sheep, goats, equine, poultry, cervids (North American deer and elk species only), and humans we used a two-step process. First, for each of the 84 pathogens we used three references texts, Diseases of Swine (Zimmerman et al. 2012), Infectious Diseases of Livestock (Coetzer et al. 2004), and Infectious Diseases of Wild Mammals (Williams and Barker 2008) to determine well-established host susceptibility using our a-priori susceptibility categories for each of the eight hosts. Second, pathogen-host combinations with susceptibilities that were identified as other than clinical or sub-clinical were then investigated using the peer-reviewed literature that included both edited book volumes and scientific journals.

To implement the second search of the peer-reviewed literature we used a combination of search terms for the scientific and common names of both hosts (Table S1) and pathogens (Table S2) to search three databases (PubMed, Scopus, and Web of Science). To capture the breadth of studies reporting the susceptibility of hosts to these pathogens all literature relating to the pathology, microbiology, epidemiology, or case reports were considered eligible and are reflected in the use of broad search terms.

Table S1. Keywords used for the eight hosts evaluated as part of this study.

Category	Common Name	Scientific Name
Swine	swine; boar; sow; pig; hog	*Suidae; Sus; scrofa*
Cattle	cattle; cow; bull	*Bovidae; Bos; taurus*
Sheep	sheep; ewe; ram	*Caprinae; Ovis; aries*
Goat	goat; billie; nannie	*Caprinae; Capra; aegagrus; hircus*
Poultry	chicken; turkey; fowl; hen; rooster	*Gallus; domesticus; Phasianidae; Meleagris; gallopavo*
Cervid	deer; white-tailed deer; mule deer; elk; wapiti	*Cervidae; Capreolinae; Odocoileus; virginianus; hemionus; Cervus; Canadensis*
Equine	horse; equine; mare; stallion	*Equidae; Equus; ferus; caballus*
Human	human	*Homo; sapiens*

Using studies identified via this second literature search host susceptibilities were categorized using the same a-priori susceptibility categories. Host susceptibilities identified during this second search were largely related to experimental hosts, rarely affected hosts, or new evidence regarding susceptibility primarily from experimental infection studies that had not been included in the references texts yet.

SUPPLEMENTARY INFORMATION

Table S2. Keywords used for swine pathogens evaluated as part of this study. All combination of terms were used.

Pathogen	Terms
Anthrax	Anthrax; Bacillus anthracis
Bovine tuberculosis	Bovine tuberculosis; mycobacterium bovis
Brucellosis (bovine)	Bovine brucellosis; Brucella abortus
Brucellosis (swine)	Swine brucellosis; brucella suis
Brucellosis (melitensis)	Brucellosis; melitensis
Hemorrhagic septicemia	Hemorrhagic septicemia; Pasteurella multocida
Leptospirosis	Leptospirosis; Leptospira
Paratuberculosis (Johne's disease)	Paratuberculosis; Johne's disease; Mycobacterium avium
Q Fever	Q Fever; Coxiella burnetii
Tularemia	Tularemia; Francisella tularensis
African swine fever	African swine fever; Asfivirus
Pseudorabies virus	Pseudorabies virus; Aujeszky's disease; Suid herpesvirus
Influenza (avian)	Avian influenza
Influenza (equine)	Equine influenza
Bluetongue	Bluetongue; Bluetongue virus
Bovine viral diarrhea virus	Bovine viral diarrhea virus
Classical swine fever	Classical swine fever; hog cholera; Classical swine fever virus
Crimean-Congo hemorrhagic fever	Crimean-Congo hemorrhagic fever virus
Eastern equine encephalomyelitis	Eastern equine encephalomyelitis virus; Triple E; sleeping sickness
Epizootic hemorrhagic disease	Epizootic hemorrhagic disease virus
Foot and mouth disease	Foot and mouth disease virus; hoof-and-mouth disease;
Heartwater	Heartwater; cowdriosis, nintas and ehrlichiosis; Ehrlichia ruminantium; Cowdria ruminantium
Infectious bovine rhinotracheitis	Infectious bovine rhinotracheitis; Bovine Herpesvirus
Japanese encephalitis	Japanese encephalitis virus
Malignant catarrhal fever	Malignant catarrhal fever; Alcelaphine gammaherpesvirus
Nipah virus encephalitis	Nipah virus encephalitis
Peste des petits ruminants virus	Peste des petits ruminants virus; ovine rinderpest
Porcine epidemic diarrhea virus	Porcine epidemic diarrhea virus
Porcine reproductive and respiratory syndrome	Porcine reproductive and respiratory syndrome virus; blue-ear pig disease
Rabies	Rabies virus
Rift Valley fever	Rift Valley fever virus
Rinderpest	Rinderpest; cattle plague; steppe murrain
Swine vesicular disease	Swine vesicular disease virus

Transmissible gastroenteritis	Transmissible gastroenteritis coronavirus
Venezuelan equine encephalomyelitis	Venezuelan equine encephalomyelitis virus
Vesicular stomatitis	Vesicular stomatitis Indiana virus; Indiana vesiculovirus
West Nile virus	West Nile virus
Trichinellosis	Trichinellosis; Trichinella
Echinococcosis	Echinococcosis; hydatid disease, hydatidosis; echinococcal disease
Leishmaniasis	Leishmaniasis; Leishmania
New world screwworm	New world screwworm; Musca macellaria
Old world screwworm	Old world screwworm; Chrysomya bezziana
Porcine cysticercosis	Porcine cysticercosis; Taenia solium; pork tapeworm
Surra (T. evansi)	Surra; Trypanosoma evansi
Trypanosomiasis (tsetse transmitted)	Trypanosomiasis; trypanosomosis

Expert review of classified host susceptibility to pathogens

Once host susceptibility was categorized for each pathogen the completed table (Table 2 in main text) was provided to five veterinary epidemiologists for review. Supporting literature including reference texts were made available during the review. Each expert reviewed the materials independently and made changes to the table based on the materials provided. Veterinary epidemiologists were selected based knowledge of human, wildlife and livestock diseases. When there was a differences in the categorization of host susceptibility the most conservative classification was used. The majority of clinical and subclinical classifications originated from the three reference texts and changes resulting from the expert review were largely restricted to experimental hosts or species that are rarely have natural infections. This final table was then used to develop transmission potential networks for the eight host species.

Supplementary Information

Detailed description of literature review used to assess current status of pathogens in North America

Data describing the current status of OIE listed pathogens in wild pigs in North America, was generated using a method to sample from the scientific literature. Our approach is based on PRISMA (Preferred Reporting Items for Systematic Reviews and Meta-Analyses) method of systematic literature review (Liberati et al. 2009, Moher et al. 2009). However our objective was not to identify all papers reporting pathogen findings but rather to generate a representative sample that could be used to determine pathogens that have existing surveillance studies. To implement this sampling we identified keywords to search three databases (PubMed, Scopus, and Web of Science) for papers reporting surveillance results, or case reports for any pathogen or disease in wild pigs in North America. In order for an article to be included the paper must have contained the terms from Table S3 in the title, key words or abstract.

Table S3. Keywords used to sample papers from the literature.

Category	Keyword List
Type	surveillance; serology; monitor; monitoring; case report; identification; pathology; microbiology; epidemiology
Species	swine; boar; pig; hog; *Sus scrofa*
Modifier	feral; wild; free ranging

We confined our search to papers with abstracts published in English since 1900. While this may have excluded some studies, particularly early studies published in Spanish or French we believe this would have been minimal for several reasons. First, free-ranging wild pigs are relatively recent new comers to Canada and most contemporary non-English journals publish abstracts in English. In addition, most Canadian wild pig populations occur in English speaking regions of Canada so we expect any excluded studies to be minimal. Second, we did not identify any studies written in Spanish with an English abstracts for studies from Mexico indicating that studies written in Spanish without English abstracts that may have been excluded were likely limited.

All scientific peer reviewed literature from Journals and edited book volumes describing any wild pig pathogens in North America was considered eligible. We excluded so called ‘gray literature’ such as government reports, technical documents or other similar documents. These documents, while potentially containing valuable data, could not be sampled effectively using our approach and may have biased our findings. In addition, the results from many of these documents likely had corresponding peer review papers.

Supplementary Information

Once all relevant sources were identified and retrieved, we reviewed each title and abstract to ensure the paper was reporting pathogen or disease findings in wild pigs in Mexico, Canada, or the United States. Our initial search identified 3,637 papers and after removal of duplicates and eligibility screening 142 papers were reviewed in detail resulting in the identification of 72 surveillance studies that were included in our study (Figure S1). The resulting papers were assumed to be a representative sample of the literature and represent the known status of pathogens in wild pigs in North America.

Figure S1. Results of systematic sampling of scientific literature.

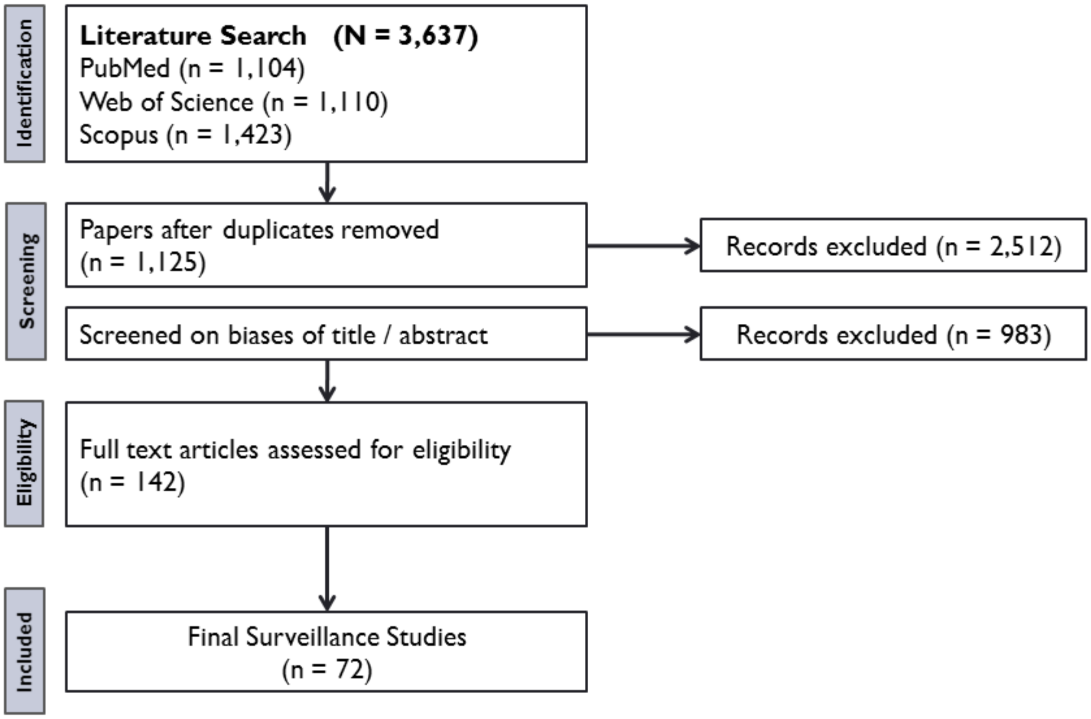

Supplementary Information

Table S4. Centrality statistics for pathogens investigated.

Pathogen		Type	Network Centrality	
			Eigenvector	Degree
Anthrax		Bacterial	1	1
Bovine Brucellosis		Bacterial	0.957	0.875
Bovine tuberculosis		Bacterial	0.957	0.875
Melitensis Brucellosis		Bacterial	0.865	0.750
Tularemia	(Vector Borne)	Bacterial	0.865	0.750
Hemorrhagic septicemia		Bacterial	0.828	0.750
Paratuberculosis		Bacterial	0.828	0.750
Swine Brucellosis		Bacterial	0.579	0.500
Rabies		Viral	0.957	0.875
Japanese encephalitis	(Vector Borne)	Viral	0.908	0.875
Venezuelan equine encephalomyelitis	(Vector Borne)	Viral	0.908	0.875
Crimean-Congo hemorrhagic fever	(Vector Borne)	Viral	0.865	0.750
Vesicular stomatitis	(Vector Borne)	Viral	0.865	0.750
Avian Influenza		Viral	0.855	0.875
Foot and mouth disease		Viral	0.820	0.750
Pseudorabies virus		Viral	0.691	0.625
Bovine viral diarrhea		Viral	0.691	0.625
Rinderpest		Viral	0.599	0.500
Nipah virus encephalitis		Viral	0.583	0.500
Infectious bovine rhinotracheitis		Viral	0.462	0.375
Equine Influenza		Viral	0.436	0.375
African swine fever	(Vector Borne)	Viral	0.170	0.125
Classical swine fever		Viral	0.170	0.125
Porcine reproductive & respiratory syndrome		Viral	0.170	0.125
Swine vesicular disease		Viral	0.170	0.125
Transmissible gastroenteritis		Viral	0.170	0.125
New world screwworm		Parasitic	1	1
Old world screwworm		Parasitic	1	1
Echinococcosis		Parasitic	0.957	0.875
Trypanosomiasis	(Vector Borne)	Parasitic	0.865	0.750
Surra	(Vector Borne)	Parasitic	0.544	0.500
Trichinellosis		Parasitic	0.436	0.375
Porcine cysticercosis		Parasitic	0.299	0.250

SUPPLEMENTARY INFORMATION

Table S5. Number of papers reporting surveillance findings for wild pigs in North America. Pathogens could be reported in more than one publication.

Pathogen	Type	OIE Listed Disease	Number of Papers Reporting
Suid herpesvirus	Virus	Yes	27
Brucella suis	Bacteria	Yes	20
Swine influenza virus	Virus		11
Porcine repro. and resp. synd. virus	Virus	Yes	10
Leptospirosis	Bacteria		8
Toxoplasma gondii	Parasite		8
Trichinella sp.	Parasite	Yes	5
Porcine circovirus type-2	Virus		5
Salmonella	Bacteria		4
Brucella sp.	Bacteria	Yes	4
Classical swine fever virus	Virus	Yes	4
Brucella abortus	Bacteria	Yes	3
Mycobacterium bovis	Bacteria	Yes	3
Mycoplasma hyopneumoniae	Bacteria		3
Nematode sp.	Parasite		3
Transmissible gastroenteritis virus	Virus	Yes	3
Vesicular stomatitis virus	Virus	Yes	3
Yersinia pestis	Bacteria		2
Actinobacillus pleuropneumoniae	Bacteria		2
Lawsonia intracellularis	Bacteria		2
Sarcocystis sp.	Parasite		2
Venezuelan equine encephalitis	Virus	Yes	2
Porcine parvovirus	Virus		2
Tularemia	Bacteria	Yes	1
Coxiella burnetii	Bacteria	Yes	1
Streptococcus suis	Bacteria		1
Mycobacterium avium	Bacteria	Yes	1
Spirometra sp.	Parasite		1
Cryptosporidium sp.	Parasite		1
Sarcoptes scabiei	Parasite		1
Haematopinus suis	Parasite		1
Neospora caninum	Parasite		1

SUPPLEMENTARY INFORMATION

References

Coetzer, J., G. Thomson, and R. Tustin. 2004. Infectious diseases of livestock. Oxford University Press Southern Africa.

Liberati, A., D. G. Altman, J. Tetzlaff, C. Mulrow, P. C. Gøtzsche, J. P. A. Ioannidis, M. Clarke, P. J. Devereaux, J. Kleijnen, and D. Moher. 2009. The PRISMA Statement for Reporting Systematic Reviews and Meta-Analyses of Studies That Evaluate Health Care Interventions: Explanation and Elaboration. PLoS Med **6**:e1000100.

Moher, D., A. Liberati, J. Tetzlaff, D. G. Altman, and P. G. The. 2009. Preferred Reporting Items for Systematic Reviews and Meta-Analyses: The PRISMA Statement. PLoS Med **6**:e1000097.

Williams, E. S., and I. K. Barker. 2008. Infectious diseases of wild mammals. 3 edition. John Wiley & Sons, Ames, Iowa, USA.

Zimmerman, J. J., L. A. Karriker, A. Ramirez, K. J. Schwartz, and G. W. Stevenson. 2012. Diseases of Swine. 10 edition. Wiley-Blackwell, West Sussex, UK.
